# Time-frequency co-movements between commodities and global economic policy uncertainty across different crises

**DOI:** 10.1016/j.heliyon.2024.e34231

**Published:** 2024-07-10

**Authors:** M. Belén Arouxet, Aurelio F. Bariviera, Verónica E. Pastor, Victoria Vampa

**Affiliations:** a Universidad Nacional de La Plata, Facultad de Ciencias Exactas, Centro de Matemática de La Plata, Argentina; b Universitat Rovira i Virgili, Department of Business, ECO-SOS, Av. Universitat 1, 43204 Reus, Spain; c Universidad de Buenos Aires, Facultad de Ingeniería, Departamento de Matemáticas, Argentina; d Universidad Nacional de La Plata, Facultad de Ingeniería, Departamento de Ciencias Básicas, Argentina

**Keywords:** Wavelet analysis, Economic policy uncertainty, Commodities, Connectedness

## Abstract

Commodity futures constitute an attractive asset class for portfolio managers. Propelled by their low correlation with other assets, commodities begin gaining popularity among investors, as they allow to capture diversification benefits. This comprehensive study examines the time and frequency spillovers between the Economic Policy Uncertainty [1] and a broad set of commodities encompassing ferrous, non-ferrous, and precious metals, food, and energy commodities over a period from December 1997 to April 2022, which includes various political, economic and health crises.

The novelty of this research lies in its extensive temporal and categorical coverage, providing an understanding of how different types of commodities respond to various crises. Furthermore, our study breaks new ground by employing wavelet analysis to gain detailed insights in both time and frequency domains in the financial time series of interest, providing a deeper understanding of the co-movements and lead-lag relationships. Specifically, we introduce the Cross Wavelet Transform (XWT) and Wavelet Coherence (WTC) analysis.

Our findings demonstrate that not all crises uniformly impact commodities. Notably, during the global financial crisis and the COVID-19 pandemic, co-movements between commodities became significantly stronger. These results highlight the heterogeneity within the commodity asset class, where individual commodities exhibit diverse underlying dynamics. Importantly, the proposed methodology facilitates the extraction of robust results even when dealing with nonlinearities and nonstationary time series data. Consequently, our work offers valuable insights for policymakers (including regulatory bodies), investors, and fund managers.

## 1 Introduction

Commodities are goods commonly used as raw materials to produce other goods and services. The pricing of commodities is heavily influenced by supply and demand interactions in the globalized world. Various factors, including weather, geopolitical events, and supply-side shocks such as wars and hurricanes, can impact supply and demand dynamics.

Traditionally, commodities have been considered real assets, whose prices are determined by their demand and the extraction and production capacity [2]. This traditional view has been changing around the new century when the number of hedge funds trading in energy markets tripled between 2004 and 2007 [3], and the traded volumes of commodity-related derivatives exceeded by twenty to thirty times the actual physical production [4]. Moreover, purchases by institutional investors increased by approximately 1200% from 2003 until 2008 [5].

In this regard, [6] argues that commodities evolved into a new investment style for institutional investors, motivated by their low or negative correlations with returns on other main asset classes. However, increased participation in portfolios leads to greater market integration and information spillovers, which can eventually reduce the benefits of diversification.

This situation makes commodities more sensible to general macroeconomic situations. As a consequence, commodities responses to general economic situations are not only of interest to traders and investors but also to governments. For example, countries like Chile heavily depend on the mining industry. Copper represents 10.9% of this country's Gross Domestic Product (GDP) and generates approximately 7.8% of the total tax revenue [7]. A stronger linkage between copper and economic uncertainty could impact the whole economy by diminishing government revenues.

Financialization, i.e. the behavior of commodities as a “finance like” product, is an elusive concept. Despite the abundant literature reporting the increasing trading volumes, and the development of new financial products constituted by commodities, the dynamics of commodities *vis-à-vis* economic variables is inconclusive. Moreover, [8] report a process of “definancialization” in energy commodities after the global financial crisis of 2008. Later, [2] reveal that such a process is also present in a broader sample of commodities. Consequently, the transition of the commodity market toward closer financial asset behavior is a contested issue.

The concept of financial and economic interconnectivity is relatively recent in economic theory. Different cutting-edge methods to identify co-movements, linkages, spillovers, coherence, or dependence between time series have been developed over the past few decades [9], [10], [11], [12], [13], [14].

Within the commodity realm, many papers deal with the study of co-movements between commodities or between commodities and traditional financial assets such as stocks or bonds. Thus, this paper aims to fill a gap in the literature by gaining insight into the return co-movements of a broad set of commodities with respect to the Economic Policy Uncertainty index developed by [1]. This information is relevant for academics, practitioners, and policymakers alike. For example, during distressed times, it could help to detect changes in portfolio diversification opportunities and portfolio optimization. Additionally, regulatory bodies could adapt their prudential regulation framework to mitigate contagion risks after a distressing event.

The Economic Policy Uncertainty index developed by [1] can be regarded as a well established metric in the economic literature, as has been recognized by more than 4700 citations in Scopus. It is demonstrated to be a versatile measure of economic uncertainty with a range of applications. Without pretending to be exhaustive, the EPU index has been used to assess its impact with respect to: (a) cross-border capital flows [15]; (b) mergers and acquisitions (at the aggregate and at the firm level) [16]; (c) daily bitcoin returns prediction [17]; (d) oil and US stock prices during the Covid-19 pandemic [18]; (e) the profitability of Ukrainian banks [19]; (f) the Turkish tourism industry [20]; (g) the firm's capital structure [21]; and (h) foreign remittances from BRIC countries [22]. These examples suggest the relevance of EPU as a reliable benchmark of economic policy uncertainty and its broad uses in diverse applications. A comprehensive literature review surrounding EPU uses can be found in [23].

From a methodological perspective, this work provides evidence of the advantages of utilizing wavelet tools for analyzing individual time series in both time and frequency domains, enabling the identification of common powers and information regarding the phase relationship between two time series.

Specifically, the data analyzed in this paper highlight the potential of wavelet tools to interpret time series quantitatively and better understand the oscillations of displacements in frequency space with other variables to find physical phase relationships. In summary, applying a wavelet-based methodology to time series is useful for understanding their dynamic relationship.

This paper contributes to the literature in four main aspects: (i) a wide sample of commodities time series during a long period (1997-2022) are studied, becoming the analysis more comprehensive than previous studies; (ii) it scrutinizes the dynamic interaction between Economic Policy Uncertainty (proxied by GEPU index) and agricultural, energy and metal commodities; (iii) the paper employs a wavelet-based analysis which is able simultaneously to study the interaction of the time series in both time and frequency; and (iv) it uncovers that different crises affect the commodity market unevenly.

The remaining of the paper is organized as follows: section 2 conducts a brief literature review; section 3 explains the methodology; sections 4 and 5 describe data and present the results and their discussion. Finally, section 6 draws the main conclusions, presents policy implications and proposes prospective research lines.

## 2 Literature review

Given the increasing importance of commodities in internationally diversified portfolios and the unique nature of this market, there has been a growing focus on studying the stochastic characteristics of commodity time series. Commodities-backed assets encompass various financial instruments that reflect agricultural, industrial, or energy-related real assets. Initially, the concept of commodities futures was to hedge risks for parties involved in the physical commodities. However, commodity financialization has altered this perspective.

The emergence of commodities as a new financial asset introduces a new style of index investing, which could affect the relationship between spot and future prices, portfolio structures, and spillovers between markets or assets. The literature surrounding these topics is inconclusive.

Whereas [24] do not find causation of commodity index investing in futures prices, [25] report no effect on volatility. The co-movements between various commodities and stocks in terms of returns or volatility have been extensively studied. For instance, [26] suggest that the co-movement between stocks, commodities, and clean energy is relatively high, while green bonds exhibit a lower correlation with other financial assets. More recently, [27] investigate the randomness of commodity prices using information theory quantifiers.

According to [28], if investors begin employing simple dynamic trading strategies, commodities futures do not offer significant diversification benefits compared to energy equities.

In [29] is shown that diversification benefits vary across time and depend on infrequent episodes. In a similar vein, [30] report that their out-of-sample results offer substantially fewer benefits than what earlier in-sample studies had revealed. Additionally, and in line with [29] portfolio gains substantially rely upon commodity and period selection.

Concurrently, [31] and [32] identify the growing relevance of linkages between agricultural and oil products using various statistical methodologies. Changes in agricultural commodities, however, across periods of economic success and upheaval are primarily due to within-sector fluctuations, rather than changes in crude oil prices.

In [33] a wavelet-based variance decomposition model is applied to the Chinese commodity market and economic policy uncertainty, finding that connectedness is high during distress periods. Similarly, [34] find, using quantile-on-quantile regression, an asymmetric and heterogeneous effect of economic policy uncertainty on commodity futures.

In [35] it is claimed that in periods corresponding to historical events (e.g. invasion of Kuwait in 1990/91, the Asian crisis in 1997/2000, the September 11th Terrorist Attack in 2001, the Iraq War in 2003, and the Global Financial Crisis in 2008) the impact of volatility on the returns of precious metals is negative and significant. In this line, [36] show significant causal impacts of the COVID-19 pandemic on the connectedness across the markets, and prove that the pandemic has been largely responsible for risk transmission across various commodity and financial markets. Recently, [37] analysed the interconnection among selected commodities during the 2008 Global Financial Crisis and the COVID-19 pandemic.

Additionally, several papers have examined the macroeconomic determinants of commodity returns or volatilities. For example, [38] suggest that gold volatility could be partially attributed to monetary variables. However, other precious metals do not exhibit this common factor, which aligns with the argument made by [39] that precious metals constitute a heterogeneous group that cannot be considered a single asset class.

Moreover, [40] identifies a significant impact of oil price shocks on the behavior of commodity sectors in China. The study also suggests that domestic macro fluctuations affect the co-movements depending on the length of the holding period. Therefore, there is a need to examine the co-movements of a wider range of commodities concerning a general economic uncertainty index. This is because, on one hand, commodity futures reflect future demand for real assets, but at the same time, they represent speculative assets.

Given the burgeoning literature surrounding intra- and inter-market spillovers in commodities and financial markets, we refer to [41] for a recent and comprehensive literature review.

## 3 Methods

The continuous wavelet transform (CWT) allows us to analyze the time series in the time-frequency domain and thus identifies localized periodicities [42]. The continuous wavelet transform of a discrete sequence $x_{n}$ is defined as the convolution of $x_{n}$ (with n=1,…,N and uniform time intervals of size *δt*) with a scaled and translated version of a wavelet function *ψ*:

(1) $$W^{X}(s,n)=\sum_{n^{\prime }=0}^{N-1}x_{n^{\prime }}\psi^{\left(⁎\right)}\left[\frac{(n^{\prime }-n)\delta t}{s}\right]$$

where (*) in Equation (1) indicates the complex conjugate and *s* is the wavelet scale. To be “admissible” as a wavelet, the function *ψ* must have zero mean and be localized in both time and frequency space. An example is the Morlet wavelet [43]. By varying the wavelet scale *s* and translating along the localized time index *n*, one can construct a picture showing the amplitude of any features versus the scale and how this amplitude varies with time. The wavelet transform $W^{X}(s,n)$ is complex if the wavelet function *ψ* is complex. Then, the transform can be divided into the amplitude $\left|W^{X}(s,n)\right|$ and the phase, $\tan^{-1}(ℜ(W^{X}(s,n))/ℑ(W^{X}(s,n)))$. The wavelet power spectrum is defined as $|W^{X}(s,n){|}^{2}$, and the cone of influence is the region of the wavelet spectrum in which edge effects become important [43]. It is the consequence of dealing with finite length time series, where errors occur at the beginning and end of the wavelet transform [43].

Wavelet analysis is also useful for analyzing two time series together, to examine whether they are somehow linked. From both CWTs, the cross wavelet transform (XWT) and the wavelet coherence (WTC) were constructed, for examining relationships in time-frequency space between two time series. These tools allow the recognition of common power and relative phase in space time-frequency, respectively, and evaluate significant coherence and confidence levels against red (Brownian) noise [43].

Given two time series $x_{t}$ and $y_{t}$ (with t=1,…,N and uniform time intervals of size *δt*), the so-called cross wavelet transform of their respective (continuous) wavelet transforms $W^{X}$ and $W^{Y}$ is a 2-D representation of complex numbers given by

(2) $$W^{XY}=W^{X}{\left(W^{Y}\right)}^{⁎},$$

and the cross wavelet power XWT is defined as $\left|W^{XY}\right|$, i.e. the modulus of Equation (2). As the XWT is calculated by multiplying the CWT of the first time series by the complex conjugate of the CWT of the second time series, its absolute value will be high in the time-frequency areas where both CWTs display high values, thus allowing the identification of common temporal patterns in the two data sets. Likewise, the phase of the complex number $W^{XY}$ gives information about the phase relation between *X* and *Y* in the time-frequency space. It is represented by the angle formed by the arrow measured counterclockwise and indicates the time delay between the two time series. For example, it will be 0° (the arrow points to the right) when the two time series are in phase, while it will be around 180° if they are in anti-phase (i.e., one reaches its maximum value when the other reaches the minimum and vice versa). For intermediate phase values, the time lag between both series can be calculated using the expression:

(3) $$\Delta t=\Delta_{\phi }\times \frac{T}{2\pi }$$

with *T* being the period and $\Delta_{\phi }$ being the gap between the two time series expressed in radians.

Another useful measure is the wavelet coherence, which is given by [43],

(4) $$R^{2}(s,n)=\frac{|S(s^{-1}W^{XY}(s,n)){|}^{2}}{S(s^{-1}|W^{X}(s,n){|}^{2})S(s^{-1}|W^{Y}(s,n){|}^{2})}$$

and is calculated by the normalized cross-correlation between *X* and *Y* time series, including a smoothing operator in both domains (time and frequency).

In other words, WTC is the result of the normalization of a smoothed version of the XWT. Its absolute value will be high (i.e., close to 1) in those areas of the temporal frequency plane where the time-frequency pattern is locally similar (hence, coherent) in the two CWTs. The interpretation of the phase information is the same as for the XWT, as they are calculated in the same way, except for the smoothing operator used in the WTC. This smoothing operation required for the WTC degrades the resolution of the result in the time-frequency domain. It can be concluded that XWT (Equation (2)) and WTC (Equation (4)) provide powerful tools for dealing with nonstationary time series. In addition, as they study simultaneously time and frequency domains, it accounts for structural breaks in the time series under scrutiny. From two CWTs, while XWT reveals high common power, WTC finds locally phase correlated behavior. As mentioned above, WTC is slightly less localized in time frequency space than the XWT. Its significance level is determined using Monte Carlo methods [43]. A software package, developed by [42], was designed to carry out XWT and WTC.

The application of the continuous wavelet transform (CWT), the cross wavelet transform (XWT) and the wavelet coherence (WTC), both to various synthetic cases and to a real case, shows the usefulness of the proposed methodology for the recognition of seasonal temporal patterns of shocks/instabilities and their relationship (time lags) with events that likely trigger them. This methodology has been previously applied in different scientific domains. For example, in [44] these tools were used successfully to interpret landslide displacement time series derived from Synthetic aperture radar interferometry (InSAR), where localized intermittent periodicities were identified. Another case is [45], which examines the power of wavelet analysis in nonlinear time series such as salinity intrusion processes. This is important because there is a lake system on the sea coast of Japan and seawater frequently intrudes into these lakes. Another application of these wavelet-based tools is presented in [46] and [47], where the COVID-19 time series for different countries were analyzed. Their analysis helped to quantify changes in the time series dynamics, including the impact of health policies and vaccination campaigns.

In this paper, we apply these wavelet-based tools to studying commodity time series in relation to a proxy of economic uncertainty. Considering the difference mentioned above between XWT and WTC, we pay attention to those regions where the graphics of XWT and WTC exhibit high power. In other words, we will work, simultaneously, with graphical representations.

**Figure 1:**
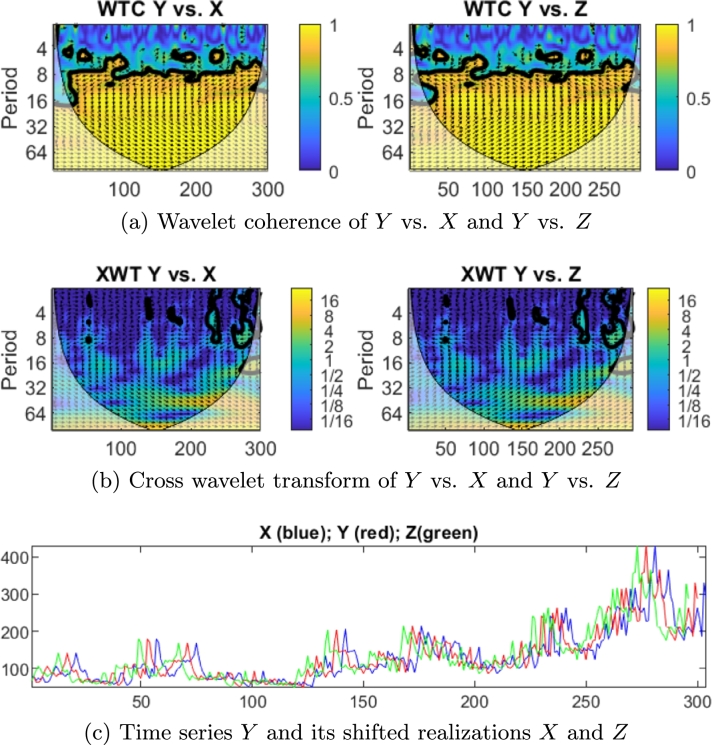
Interpretation of the lead/lag relationship according to arrows' orientation in graphics of XWT and WTC, for any time series *Y*, and its shifted realizations *X* and *Z*, for *s* = 4 periods.

The interpretation of the lead/lag relationship of two time series, related to the orientation of the arrows is as follows. Let's consider a time series *Y*. We construct series *X* and *Z*, which are shifted realizations of *Y*, such that $X_{t}=Y_{t-s}$ and $Z_{t}=Y_{t+s}$. Thus, *Y* lags behind *X* and *Y* leads *Z*, as displayed in Fig. 1c. We construct WTC and XWT graphs of *Y* vs. *X* and *Y* vs. *Z*, as shown in Fig. 1a and Fig. 1b, respectively. It can be observed that when the arrows point upwards (1st and 2nd quadrants) the first series leads the second and vice versa.

### 3.1 Application: crude oil analysis

In this section we perform a detailed analysis of the crude oil and GEPU time series, encompassing the information obtained by the continuous wavelet power spectrum (given by the CWT), the cross wavelet transform and wavelet coherence.

**Figure 2:**
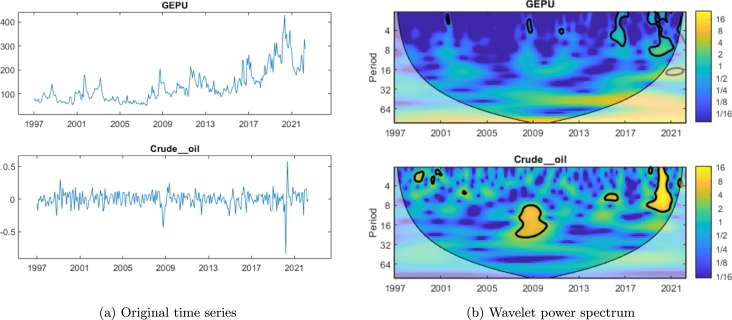
Crude oil returns and GEPU time series (a) and their continuous wavelet power spectrum.

Fig. 2 shows GEPU and crude oil time series (2a) and their power spectra (2b) provided by the respective wavelet transforms. In yellow and light green/blue, we observe the areas where wavelet power is significant. Regarding crude oil, we observed a peak in the 8-16 period around 2009, and another one in the 1-8 period in 2020. Whereas, for GEPU, a less (but significant) peak in the 1-4 period in 2017 and 2020 can be observed.

**Figure 3:**
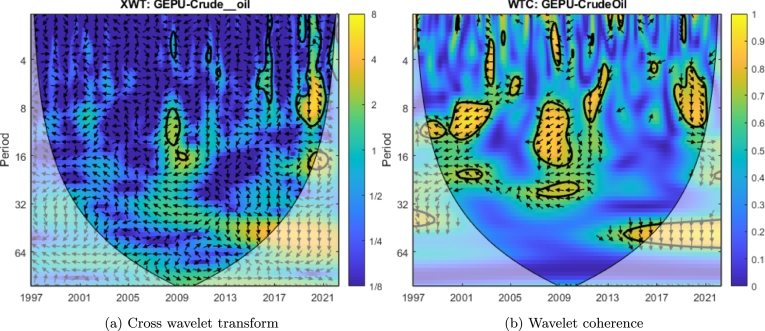
Cross wavelet transform (left) and wavelet coherence (right) of the standardized crude oil and GEPU time series.

Fig. 3a shows the cross wavelet transform (XWT) and depicts the areas with high common power between GEPU and crude oil. Such areas appear in warm colors (especially yellow) and are contoured by thick black lines, conforming to a closed region. This figure reveals that crude oil and GEPU share significant power for the 8-14 period in 2008-2009, for the 2-10 period in 2020, and (to a lesser extent) for the 2-9 period in 2017. The arrows' angles indicate the phase delay. These phase angles are not constant across scales, a signature of varying time lag in signal propagation.

Finally, Fig. 3b shows the WTC, which represents the coherence in the time-frequency space of both signals. There we observe a significant coherence in the 8-16 period around 2008-2009 and the 4-10 period around 2020. Our analysis encompasses the results of both the XWT and the WTC. In other words, we consider the significant coherent areas, only when XWT is also significant. Therefore, we will consider (for these two-time series) only the coherence and delay in 2008-2009 and 2020.

If we focus our attention on the years 2008-2009, considering the overlapping areas of XWT and WTC and the arrows' orientation, the phase delay between GEPU and crude oil (following Equation (3)) is between 5 to 6 months for high-frequency analysis and between 6 to and 10 months for low-frequency analysis. Additionally, if we now consider the year 2020, the analysis reveals a phase delay between both time series between 5 to 6 months at high frequency and between 6 to 8 months at low frequency. In all cases, GEPU lags crude oil for the mentioned delay periods.

## 4 Data and results

This paper uses monthly data on 16 commodities and GEPU index, spanning from January 1997 to April 2022. Each time series comprises a total of 304 observations. Selected commodities encompass ferrous, non-ferrous, and precious metals, food, and energy commodities: copper, aluminium, nickel, silver, gold, palladium, platinum, cotton, wheat, cocoa, coffee, raw sugar, corn, soyabeans, crude oil, and heating oil. Commodities' data were downloaded from Eikon and GEPU index from its website. The descriptive statistics of these time series are displayed in Appendix A (Table A.3).

The Global Economic Policy Uncertainty (GEPU) index, developed by [1] and [48], provides a quantitative measure of economic and policy uncertainty on a global scale. It achieves this by aggregating national Economic Policy Uncertainty (EPU) indices for 21 countries, which account for roughly 71% of global output (adjusted for Purchasing Power Parity). The methodology behind GEPU index is based on the analysis of newspaper coverage. Each national EPU index is constructed by examining the relative frequency of articles containing terms related to the economy, policy, and uncertainty. This frequency is a proxy for public and business concerns surrounding government actions that might impact the economic landscape. The key steps involved in its elaboration are the following:

(a) Newspaper Search: A comprehensive search is conducted across major newspapers in each of the 21 countries. The search targets articles containing a predefined set of terms that pass the Economic Uncertainty filter: (economic OR economy) AND (uncertain OR uncertainty).
(b) Frequency Analysis: The frequency of these terms appearing together within the same article is then analyzed. A higher co-occurrence suggests a greater focus on economic policy uncertainty within the media coverage. This process undergoes human auditing to ensure the accuracy and robustness of the index.
(c) National EPU Index Construction: Based on the frequency analysis, an EPU index is calculated for each country.
(d) Global GEPU Index Construction: the national EPU indices are combined to create GEPU index. This is achieved by calculating a GDP-weighted average.

Further details about the methodology and countries covered by individual EPU indices can be accessed on the official website of the index https://www.policyuncertainty.com/index.html

Different crises and stressful situations cross the period under study. These events are assumed to be captured by GEPU index. Due to their different nature, intensity, and particular characteristics, we could expect commodities to react differently to uncertainty. That is why our analysis has proposed to distinguish and cluster different behaviors, classifying them according to the most important crises identified in the period examined. We select three important crises, each of them of a different nature. The first is the 2001 dot-com crisis, corresponding to a technology-inspired boom in the late 1990s. The crisis is reflected in the sustained downturn of the NASDAQ composite index from its peak in March 2000 and the decreases were later accentuated by the events of September 11. As such, it can be considered a crisis in a specific sector of the economy, aggravated by a political event. The second is the 2008 Global Financial Crisis, the first worldwide crisis of the 21st century, which affected many countries. Finally, the third is the COVID-19 pandemic. Unlike previous events, this could be considered an unforeseeable, once-in-a-lifetime event. Despite primarily being a health emergency, it had significant economic effects, including supply chain disruptions and a sudden halt in many economic activities worldwide. Thus, our analysis precisely sheds light on the various linkages between GEPU and commodities during these events.

Table 1 and Table 2 summarize the lead-lag relationships and the delay between GEPU and each one of the commodities in the three selected crises. We consider a significant relationship only in those regions where the graphics of XWT and WTC exhibit high power. In addition, as explained in detail in section 3, the delay is measured by the angle of the arrows in WTC graphics.

**Table 1:**
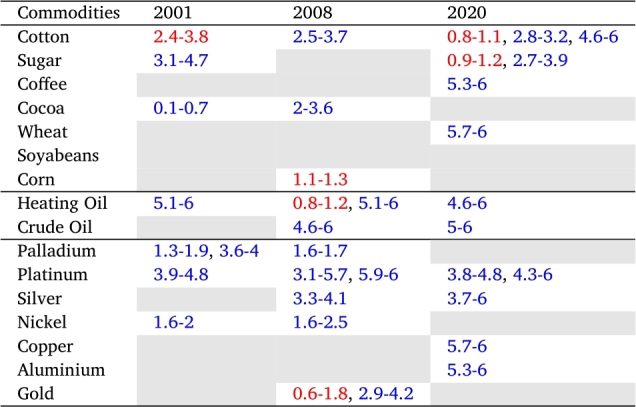
Lead-lag relationship (in months) between a given commodity and GEPU, considered at high frequency (less than 6 months). Colors indicate the sense of the relationship: red means that GEPU lags the commodity, whereas blue signals that GEPU leads the commodity.

**Table 2:**
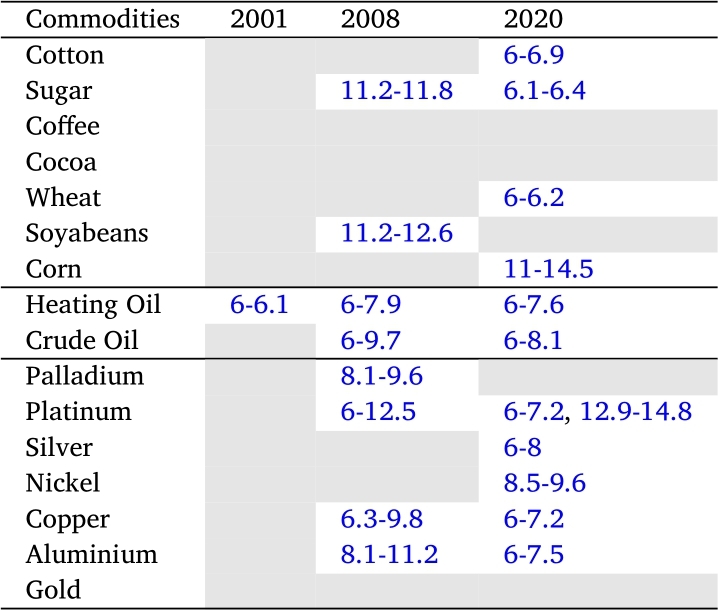
Lead-lag relationship (in months) between a given commodity and GEPU, considering at low frequency (greater than 6 months). Colors indicate the sense of the relationship: red means that GEPU lags the commodity, whereas blue signals that GEPU leads the commodity.

### 4.1 The dot-com crisis

In the year 2001, two events converge. On one side the burst of the so-called dot-com bubble and the 9/11 attack. In addition to a profound downturn in the NASDAQ Composite Index, it produced a reduction in the venture capital available for financing innovative firms, and a change in the mentality of CEOs and investors, regarding tech firms. Moreover, the September 11 attacks contributed further to an increase in global and political uncertainty.

**Figure 4:**
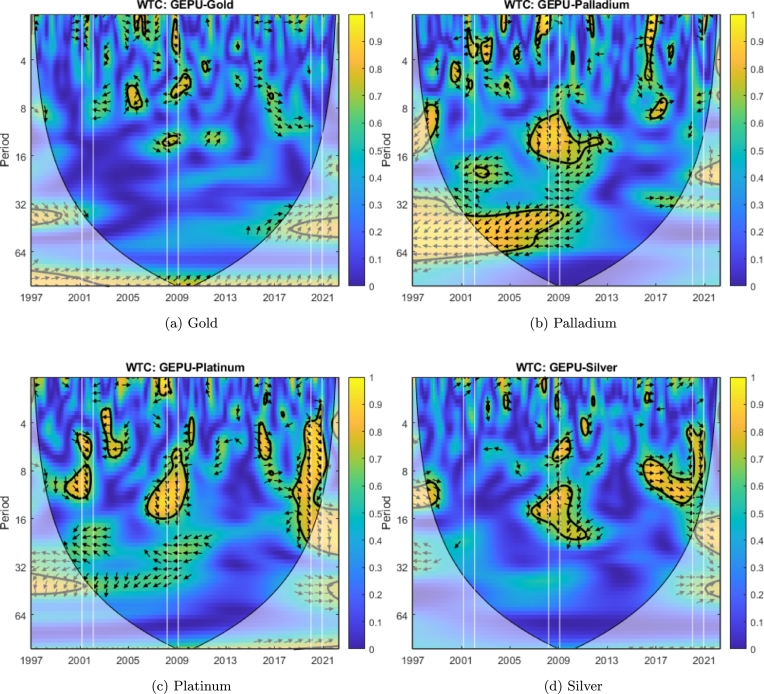
Wavelet Coherence (WTC) between GEPU and precious metals.

**Figure 5:**
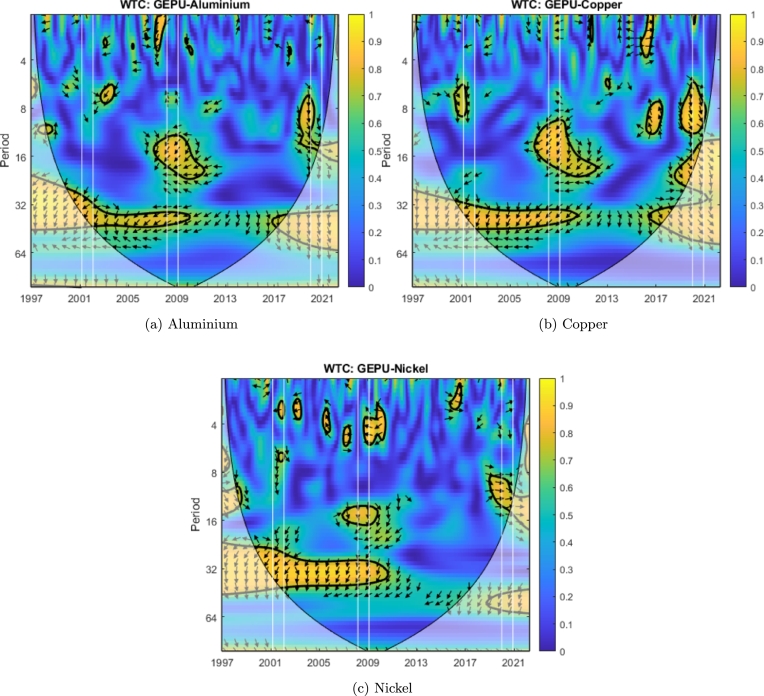
Wavelet Coherence (WTC) between GEPU and non-precious metals.

**Figure 6:**
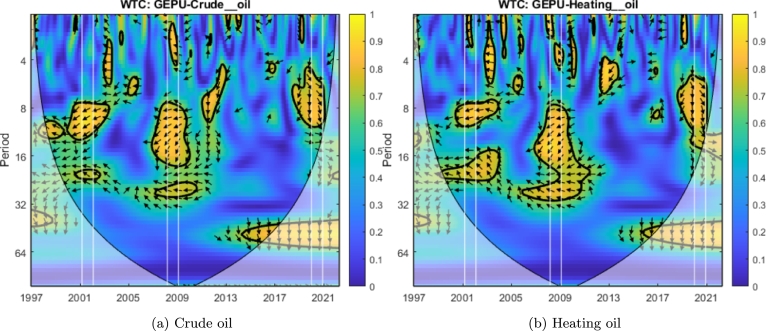
Wavelet Coherence (WTC) between GEPU and energy.

**Figure 7:**
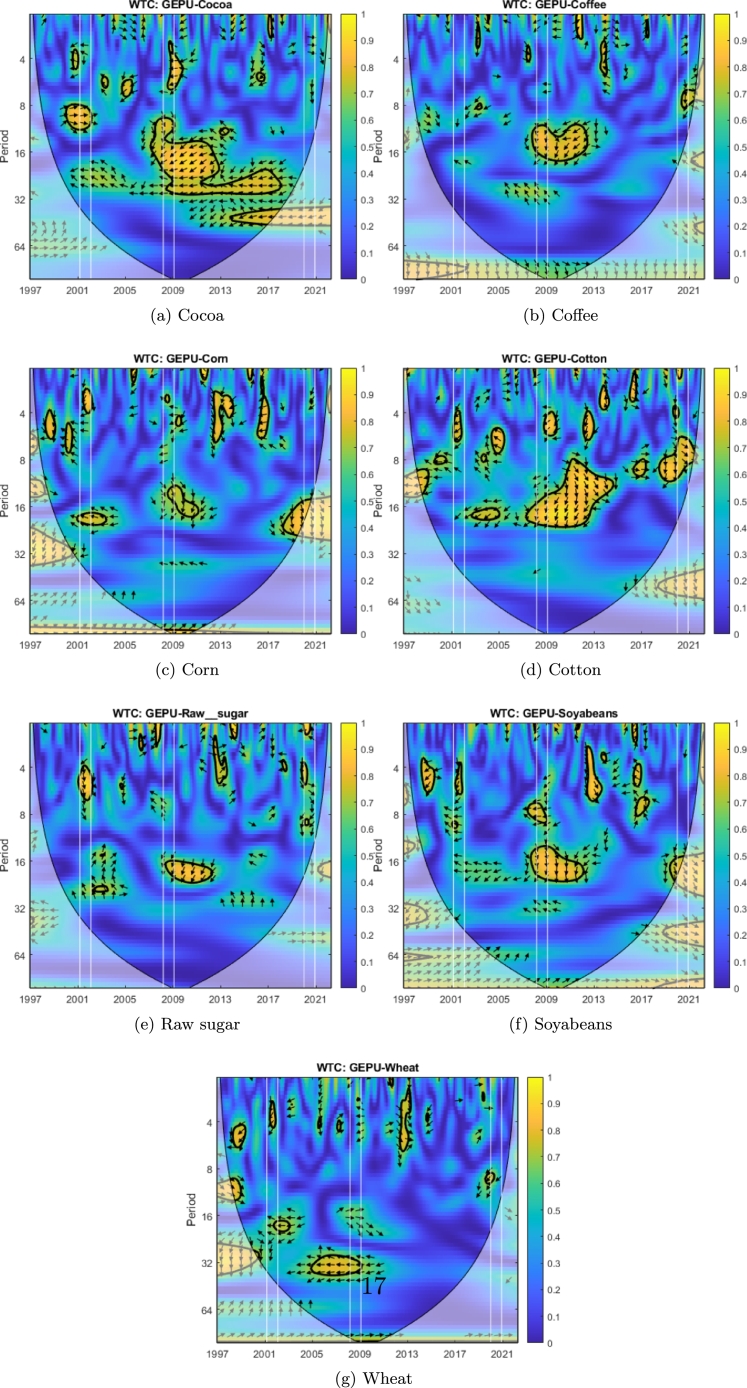
Wavelet Coherence (WTC) between GEPU and agricultural commodities.

We can observe that cotton (Fig. 7d), sugar (Fig. 7e), palladium (Fig. 4b), platinum (Fig. 4c), and nickel (Fig. 5c) exhibit coherence with GEPU at high frequency (T=2−7) with delays between 1 to 5 months. Additionally, heating oil (Fig. 6b) has coherence with GEPU at lower frequencies (T=8−11) with delays of up to 6 months. This feature is also valid for cocoa (Fig. 7a) but with a shorter lead-lag relationship. There are no intersection areas for the remaining commodities between WTC and XWT. This period is characterized by short-term co-movements between GEPU and certain commodities, particularly at higher frequencies (less than 6 months). In contrast, the co-movements also extended to lower frequencies during other crises.

### 4.2 The 2008 financial crisis

The 2008 Global Financial Crisis (GFC) is undoubtedly the most important distress event in the 21st century. From the comparison of results shown in Figure 6, Figure 7 and in Table 1, Table 2, the wavelet coherence of GEPU with respect to the commodities can be observed. Cotton (Fig. 7d), cocoa (Fig. 7a), corn (Fig. 7c), silver (Fig. 4d), nickel (Fig. 5c), and gold (Fig. 4a) show coherence at high frequencies (T=2−7), with a lag of about 4 months. On the other hand, sugar (Fig. 7e), soybeans (Fig. 7f), copper (Fig. 5b), and aluminium (Fig. 5a) show common behavior at low frequencies (T=10−19), with a lag of 6 to 13 months. Crude oil shows a coherence with GEPU at low frequencies (T=8−14), with a lag between 5 to 9 months, while heating oil, palladium, and platinum show coherence at both high (T=2−8), and low frequencies (T=8−16), with lags ranging from 5 to 12 months.

### 4.3 COVID-19 pandemic

Encompassing the visual analysis of Figure 6, Figure 7 and the summarized information in Table 1, Table 2, we detect that cotton (Fig. 7d), sugar (Fig. 7e), heating oil (Fig. 6b) and silver (Fig. 4d) show coherent behavior with GEPU at high frequencies (T=2−7) with delays less than 5 months. Besides, cotton (Fig. 7d), sugar (Fig. 7e), coffee (Fig. 7b), wheat (Fig. 7g), heating oil (Fig. 6b), crude oil (Fig. 6a), palladium (Fig. 4b), silver (Fig. 4d), copper (Fig. 5b), nickel (Fig. 5c), and aluminium (Fig. 5a) display coherent behavior at lower frequencies (T=7−10) with delays between 5 to 8 months, except for nickel (Fig. 5c), whose delay is around 9 months. Moreover, corn (Fig. 7c) displays coherence at even lower frequencies (T=14−19) with delays between 11 to 15 months. Finally, platinum (Fig. 4c) features both high (T=3−10) and low (T=16−19) frequency coherence, with delays of 3 to 7 and 13 to 15 months, respectively.

## 5 Discussion

Based on the results described in the previous section, we analyze and compare the relationships between commodities and crises globally. We also benchmark our results with those obtained by other authors.

At a general level, and looking only at the WTC, crude-oil and heating oil present very significant and similar regions during the three crises (considering the amount colored in yellow). In the case of corn, raw sugar, and wheat, the WTC show similar behavior in the three crises but is much less significant than the energy commodities. In the case of cotton and platinum, the WTC figures are similar (with medium significance) during the three crises. Regarding the rest of the commodities, the WTC figures are much more significant in the 2009 crisis than in the other two. In summary, there are asymmetric, time-varying linkages between commodities and GEPU, that cannot be attributed to commodity classes.

We summarize our findings in Fig. 9. Each set of the Venn diagram represents one of the crises considered, and the names of the commodities are those that exhibit coherence with GEPU during the respective crisis.

We observe that the Global Financial Crisis (GFC) of 2008 and the COVID-19 crisis were the most significant events in our sample. During these events, 16 commodities were affected by economic uncertainty, whereas only 7 commodities were affected by the 2001 crisis.

The composition of commodities most affected by the various crises differed significantly. It must be highlighted that the GFC was the single crisis where gold dynamics reflected some commonality with GEPU, albeit during a very short time. During the 2001 crisis, GEPU exhibits coherent behavior with some agricultural commodities and metals. However, neither important agricultural products such as wheat and soyabeans, nor precious metals such as gold and silver were affected.

Finally, gold is mainly detached from the evolution of the general economy. Furthermore, coherence was detected only, for a short period, during the GFC, as can be seen in Table 1. Also, soyabeans were only affected by the GFC, but for a longer period (almost 13 months). The only commodities that do not display co-movements with GEPU are wheat and coffee. Unlike previous crises, the COVID-19 emergency did not produce a coherence between GEPU and gold, palladium, soyabeans, or cocoa. This could be due to the quick reaction of different governments, providing financial aid and subsidies to individuals and firms, to alleviate the effects of lockdowns and possible economic downturns.

[49] documented a strong relationship between fuel prices and commodity prices (industrial, food, metal), employing various methods, including wavelet coherence. In contrast to their findings, we observe that this correlation is primarily evident during crisis periods.

**Figure 8:**
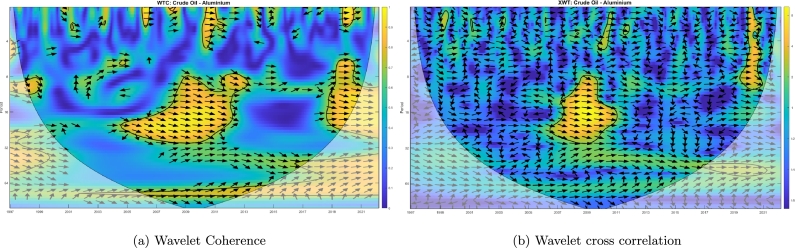
Wavelet Coherence (WTC) and Wavelet cross correlation (XWT) between crude oil and aluminium.

If we analyze the coherence between crude oil and aluminium (see Fig. 8a), after accounting for the wavelet cross-correlation (Fig. 8b), the only areas with significant coherence correspond roughly to 2008 and 2020. Contrary to [49], [50] using cross spectral quantile and network connectivity spillover approaches, they observe no significant short- and medium-term spillovers between different commodity classes. They only report some traces of spillover from crude oil and Brent oil to aluminium and from silver to copper. Consequently, they argue that the consistency across commodity classes remains minimal for several market commodities and that within-group consistency is low to moderate.

**Figure 9:**
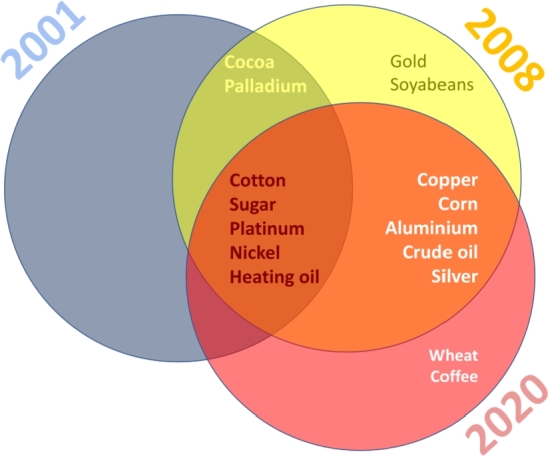
Venn diagram of the significant coherence between GEPU and each of the commodities, during the three crises considered.

In their comprehensive literature review, [41] find that intra- and inter-connectedness in commodities are sparked in crises. Our paper complements and refines those results. We detect heterogeneity in the commodity market, reflected in the different coherence between each commodity in our sample and GEPU. In this sense, the traditional view of the commodity market as a uniform body with similar stochastic behavior is challenged. On the contrary, our findings indicate that there is no such thing as a “commodity market” but rather a heterogeneous mosaic of assets under an umbrella name, whose dynamics are not identical. The coherence between GEPU and each commodity could hardly fit into broad category labels. In other words, co-movements are not the same across all metals, agricultural or energy commodities.

Our findings are consistent with those of [51], who find that structural breaks in commodity return volatility are more idiosyncratic, rather than category-wide. Thus, they corroborate our assessment that commodities are too diverse to be considered a single asset class.

[52] identified a significant increase in average connectivity, from 15% before the Global Financial Crisis (GFC) to 50% after 2008. We concur with the authors that the 2008 GFC significantly impacted the international economic system, a trend reflected in our findings as well. However, we note no strong correlation during the inter-crisis period.

[53] conducted a study akin to ours, employing a similar methodology and approach, examining the relationship between economic uncertainty stemming from the COVID-19 pandemic and its impact on the industrial sector, compared with the effects of the GFC in the United States. However, our results differ from theirs, in the sense that not all commodities reacted equally to the pandemic and the coherence periods are generally shorter concerning the GFC.

## 6 Conclusions

This study offers a comprehensive analysis of a diverse array of commodities, setting it apart from prior research. It contributes to the literature by offering a detailed and general analysis of the interplay between economic policy uncertainty and commodity markets, highlighting the distinct behavior of commodities in different crisis contexts.

This paper discussed the time-frequency relationship between GEPU and different commodities, using an advanced quantitative technique, namely Wavelet analysis. As mentioned in the previous section, and in line with our results and the previous literature, the interaction between commodities and the overall economic environment is not a settled issue. This study offers a comprehensive analysis of a diverse array of commodities, setting it apart from prior research. Another novelty of this paper, from a methodological point of view, is the simultaneous study of the Cross Wavelet Transform (XWT) and the Wavelet Coherence (WTC), who provide complementary information about the involved time series. Policymakers could incentivize this kind of interdisciplinary research to examine the interactions between commodity markets, geopolitical uncertainty, climate risk, and other economic and financial factors. This could inform the design of more effective policies for managing associated risks.

Our analysis reveals that not all crises affect commodities in the same way. Even though commodities are usually studied as a unified asset class, they are heterogeneous financial assets, whose underlying dynamics are different from one another. This was reflected in the uneven response to different crises. The GFC stands out as the event exhibiting the strongest coherence between GEPU index and commodity prices.

Understanding the return dynamics of commodities and their behavior across various crises is crucial for a diverse range of stakeholders and has important policy implications. As in the case of the banking sector, where the Basel accords set a framework for banking supervision and regulation worldwide, commodities markets could also be subject to an international agreement. Regulatory bodies could explore the development of early warning systems that monitor GEPU and specific commodity price movements to identify potential financial instability. As it is recognized in [54]: “The use of corn for biofuel production can compete with the production of food crops, leading to potential food price increases and shortages in some areas”. Thus, distressed commodity markets could disrupt the global economy, particularly for food-producing developing countries. Consequently, policymakers could promote international cooperation in market regulation to mitigate the cross-border transmission of crises. By working closely with other countries and international organizations, consistent standards and regulations could be developed to contain contagion and manage global economic shocks effectively. The varied responses from commodities to different crises types imply a need for flexible market regulations that can be adapted based on the nature of the crisis. Encouraging the development of more sophisticated risk management mechanisms, including innovative financial instruments and insurance systems tailored to address volatility risks associated with commodity price fluctuations during crises, could enhance market resilience. Adopting a risk-based approach to regulation could also be beneficial, wherein stricter controls might be implemented for commodities exhibiting stronger co-movements with GEPU during specific kinds of crises, to mitigate contagion across markets.

Additionally, fostering transparency and enhancing information disclosure practices within commodity markets could empower investors to make more informed decisions during crisis periods. Investors can construct efficient portfolios based on a correct assessment of risk associated with different crises. Hedgers could fine-tune their long and short positions to offset price risk.

This study has some limitations. First, we focus solely on a broad range of commodities. Thus, this topic will deserve further research. Expanding the analysis to include additional asset classes, such as stocks, green bonds, and digital assets, could provide a more comprehensive picture of how financial markets interact with overall economic activity, especially during crises. Second, we utilize monthly data due to the frequency of GEPU index. While this reduces noise in the time series of uncertainty and commodity prices, it limits the ability to examine responses to more localized events. If studying such events is of research interest, employing a high-frequency uncertainty proxy would be necessary. Candidate alternatives include the Geopolitical Risk Index by [55] or the Google Trends Uncertainty Index by [56], though the latter is limited to the United States. Additionally, the impact of novel events, such as the recent conclusion of quantitative easing policies by the Federal Reserve and European Central Bank, is not covered in our sampling period and warrants future investigation.

## CRediT authorship contribution statement

**M. Belén Arouxet:** Writing – original draft, Visualization, Software, Methodology, Formal analysis, Data curation. **Aurelio F. Bariviera:** Writing – original draft, Supervision, Resources, Investigation, Conceptualization. **Verónica E. Pastor:** Writing – original draft, Visualization, Software, Methodology, Investigation, Data curation. **Victoria Vampa:** Writing – original draft, Supervision, Methodology, Formal analysis, Conceptualization.

## Declaration of Competing Interest

The authors declare the following financial interests/personal relationships which may be considered as potential competing interests: Aurelio F. Bariviera is Associate Editor of Heliyon Finance. If there are other authors, they declare that they have no known competing financial interests or personal relationships that could have appeared to influence the work reported in this paper.

## Figures and Tables

**Table A.3:** Descriptive statistics of the commodities and GEPU.

Time series	# Obs.	Mean	Median	Std. Dev.	Min	Max	Skewness	Kurtosis	Jarque-Bera
Copper-LME U$/MT	304	5177.184	5802.000	1341.250	10343.000	2632.942	-0.064	1.729	20.665
Aluminium 99.7%-LME U$/MT	304	1891.089	1795.000	1150.000	3501.000	457.503	0.864	3.241	38.579
Nickel - LME U$/MT	304	14549.556	13544.625	4009.500	50575.000	7933.600	1.534	6.535	277.505
Silver U$/Troy OZ	304	14.556	14.630	4.190	46.560	8.801	0.779	3.234	31.456
Gold Bullion LBM $/t oz	304	936.729	980.025	254.000	1976.520	535.846	0.143	1.636	24.617
Palladium U$/Troy Ounce	304	704.494	557.000	116.500	2981.000	606.620	1.847	5.895	278.947
Platinum London Free Market $/Troy oz	304	992.282	942.000	336.000	2174.000	418.884	0.334	2.404	10.132
Cotton,1 1/16Str $/Lb	304	0.668	0.640	0.280	1.910	0.229	2.139	10.611	965.660
Wheat No.2, Soft Red U$/Bu	304	4.693	4.385	2.000	11.500	1.778	0.685	3.028	23.777
Cocoa-ICCO US$/MT	304	2147.991	2157.855	800.390	3634.650	680.794	0.000	2.149	9.184
Coffee-ICO Composite Daily ICA c/lb	304	111.923	111.650	41.960	247.390	41.323	0.466	3.289	12.077
Raw Sugar-ISA c/lb	304	13.555	12.830	4.740	31.260	5.496	0.682	3.072	23.606
Corn No.2 Yellow U$/Bushel	304	3.563	3.345	1.470	8.130	1.594	1.014	3.182	52.541
Soyabeans No.1 Yellow $/Bushel	304	8.937	8.715	4.100	17.780	3.332	0.442	2.256	16.918
Crude Oil-WTI-U$/BBL	304	56.439	53.775	11.130	140.970	28.110	0.363	2.240	14.010
Heating Oil NY No. 2 U$/GAL	304	1.651	1.669	0.307	3.928	0.850	0.329	2.164	14.348
GEPU	304	131.854	111.450	48.875	430.349	69.397	1.420	4.912	148.423

## Data Availability

The data supporting this study are available from the corresponding author upon reasonable request.
